# Turning up the heat on COVID-19: heat as a therapeutic intervention

**DOI:** 10.12688/f1000research.23299.2

**Published:** 2020-07-20

**Authors:** Marc Cohen

**Affiliations:** 1 Extreme Wellness Institute, Melbourne, VIC, Australia

**Keywords:** Heat stress, hyperthermia, sauna, steam inhalation, balneotherapy, COVID-19

## Abstract

Enveloped viruses such as SAR-CoV-2 are sensitive to heat and are destroyed by temperatures tolerable to humans. All mammals use fever to deal with infections and heat has been used throughout human history in the form of hot springs, saunas, hammams, steam-rooms, sweat-lodges, steam inhalations, hot mud and poultices to prevent and treat respiratory infections and enhance health and wellbeing. This paper reviews the evidence for using heat to treat and prevent viral infections and discusses potential cellular, physiological and psychological mechanisms of action. In the initial phase of infection, heat applied to the upper airways can support the immune system’s first line of defence by supporting muco-ciliary clearance and inhibiting or deactivating virions where they first lodge. This may be further enhanced by the inhalation of steam containing essential oils with anti-viral, mucolytic and anxiolytic properties. Heat applied to the whole body can further support the immune system’s second line of defence by mimicking fever and activating innate and acquired immune defences and building physiological resilience. Heat-based treatments also offer psychological benefits and enhanced mental wellness by focusing attention on positive action, enhancing relaxation and sleep, inducing 'forced-mindfulness', and invoking the power of positive thinking and ‘remembered wellness’. Heat is a cheap, convenient and widely accessible therapeutic modality and while no clinical protocols exist for using heat to treat COVID-19, protocols that draw from traditional practices and consider contraindications, adverse effects and infection control measures could be developed and implemented rapidly and inexpensively on a wide scale. While there are significant challenges in implementing heat-based therapies during the current pandemic, these therapies present an opportunity to integrate natural medicine, conventional medicine and traditional wellness practices, and support the wellbeing of both patients and medical staff, while building community resilience and reducing the likelihood and impact of future pandemics.

## Heat in viruses and mammals

Life exists within a narrowly defined temperature range, yet viruses, which are not technically alive, can remain biologically active in a wide range of environments. Enveloped viruses, such as rhinoviruses and coronaviruses, are most active in cool dry conditions, which are associated with increased occurrence of respiratory tract infections ( Mäkinen *et al.,* 2009), including infections with SARS-CoV ( Chan *et al.,* 2011) and SAR-CoV-2 ( Sajadi *et al.,* 2020; Wang *et al.*, 2020). While enveloped viruses can remain active for long periods in cold conditions, their lipid envelopes are destroyed by temperatures tolerable to humans. The heat sensitivity of viruses is used routinely to deactivate viruses within vaccines, and temperatures of 55 to 65°C for 15 to 30 minutes are reported to deactivate a range of enveloped viruses, including coronaviruses ( Darnell *et al.,* 2004; Duan *et al.,* 2003; Hu *et al.,* 2011; Kampf *et al.,* 2020; Lelie *et al.,* 1987; WHO Report, 2003).

The first line of defence against respiratory viruses is the nasal cavity and sinuses, which maintains a protective mucosal barrier that allows viruses to be trapped, identified by the immune system and swept away, as well as serving an important thermoregulatory role. The nasal cavity and sinuses in humans constantly exchanging heat with inhaled air through convection, conduction and evaporation, which serves to cool inhaled air in summer and warm and humidify air in winter ( Soni & Nayak, 2019). The upper airways also serve as the first line of defence against respiratory viruses by maintaining a protective mucosal barrier that filters and traps foreign particles and pathogens in a layer of watery mucus, and enables them to be identified by the immune system. This mucus is then moved by cilia towards the pharynx, where it is either swallowed or expelled by coughing, sneezing and nose blowing. A moist mobile mucosal barrier is vital in the defence against respiratory infections and this barrier is enhanced by warm humid conditions and impaired by cigarette smoke and particulate pollution ( Fahy & Dickey, 2010).

In winter when sunlight is restricted and the air is cold and dry, the nasal cavity becomes the coldest part of the body and if the airways dry out and the mucous becomes thicker and more difficult to clear, conditions become more favourable for viral penetration and replication. The ability of cool, dry conditions to enhance viral infection has been demonstrated in mice, with humidity of around 20% being shown to slow muco-ciliary clearance, impair innate antiviral defence, and reduce tissue repair function, leading to more rapid and severe illness compared with humidity of 50% ( Kudo *et al.,* 2019).

If respiratory viruses get past the first line of defence, fever is produced as part of the acute phase response, which forms the immune system’s second line of defence. Fever is a cardinal response to infection that has been conserved within vertebrates for more than 600 million years. Ectotherms as diverse as reptiles, fish, and insects raise their core temperature during infection through behavioural regulation, and all mammals have evolved sophisticated mechanisms to create and disperse heat and manage the oxidative stress associated with operating at higher temperatures ( Evans *et al.,* 2015).

## Heat as medicine

The use of heat for cleansing and healing is a conscious extension of mammals’ use of heat that has been practiced throughout human history. Hot springs, saunas, hammams, steam rooms, sweat lodges, steam inhalations, baths, hot mud and poultices have been used traditionally in cold, dry climates to prevent and treat respiratory infections and to enhance overall health and wellbeing. Heat-based therapies are not widely used in mainstream medicine, other than the local application of hot packs for symptomatic relief, or the use of novel technologies such as microwaves, radiofrequency energy, ultrasound, infrared radiators, ferromagnetic seeds, nanoparticles and resistive implants that apply heat to treat various cancers ( Chicheł *et al.,* 2007). Heat-based treatments however, are standard offerings in wellness establishments, such as hot springs, bathing facilities, gymnasiums, fitness centers, hotels and resorts, where they are used for both therapy and recreation ( Clark-Kennedy & Cohen, 2017).

There are multiple lines of evidence to support the use of heat and humidity for the prevention and treatment of viral respiratory infections. Historical and emerging evidence suggests regular sauna bathing enhances cardiovascular, respiratory and immune function as well as improving mood and quality of life ( Hussain & Cohen, 2018)( Laukkanen *et al.,* 2018). Finnish sauna bathing, which involves brief exposures to high environmental temperature (80°C-100°C) has been shown to reduce the risk of all-cause mortality, sudden cardiac death, cardiovascular disease and vascular diseases such as high blood pressure and stroke, along with the risk of neurocognitive diseases, skin conditions and painful conditions such as rheumatic diseases and headache ( Laukkanen *et al.,* 2015; Laukkanen *et al.,* 2018; Laukkanen & Kunutsor, 2019). Epidemiological evidence further suggests that frequent sauna bathing is associated with a reduced risk of pneumonia and viral infection ( Kunutsor *et al.,* 2017a) ( Kunutsor *et al.,* 2017a; Kunutsor *et al.,* 2017b), and randomised controlled trial evidence suggests that regular saunas can halve the incidence of respiratory viral infections ( Ernst *et al.,* 1990). Randomised controlled trials further suggest hot air can treat respiratory infections with humidified air at temperatures above 43°C for 20 to 30 minutes being found to reduce viral shedding, provide immediate relief of symptoms and improve the course of the common cold ( Tyrrell, 1988; Tyrrell *et al.,* 1989).

## Mechanisms of action

The mechanisms by which heat overcomes viral infections depends on the setting, source, temperature, humidity, location and time course of applied heat. Whether internally generated or externally applied, heat has a profound influence on host defences and physiological resilience, as well as on viral load and virulence, and engages adaptive thermoregulatory mechanisms that can increase or decrease body temperature in order to restore homeostasis ( Schieber & Ayres, 2016).

Hyperthermia and heat stress have multiple actions that may help mitigate viral infections. These include direct inhibition of pathogens, stimulation of both the innate and adaptive arms of the immune system and activation of regulatory processes that dampen inflammatory responses and prevent excessive tissue damage ( Evans *et al.,* 2015). Inhalation of hot air supports the immune system’s first line of defence by direct inhibition or deactivation of virions in the upper airways where they first lodge. Inhalation of hot humid air also supports muco-ciliary clearance, which can be further enhanced by inhalation of steam ( Gujrathi *et al.,* 2016). Heat applied to the whole body further supports the immune system’s second line of defence by inducing heat-stress that mimics the effects of fever ( Schieber & Ayres, 2016).

Febrile temperatures activate multiple cellular responses that include complex reciprocal regulation between immune system activation, inflammation and the heat shock response pathway ( Singh & Hasday, 2013). While the mechanisms by which heat-stress modulates immune function are not fully understood, higher temperatures have been shown to activate immune cells by making their cell membranes more fluid, which increases cell differentiation and activation by viral antigens and enables a faster and more effective response to viral threats ( Mace *et al.,* 2011).

Acute heat stress has been shown to increase the TNF-alpha response of monocytes ( Zellner *et al.,* 2002), enhance interleukin-2 induced activity of Natural Killer (NK) cells ( Kappel *et al.,* 1991), and cause a 10-fold increase in interferon-γ production by T-lymphocytes ( Downing *et al.,* 1988) while regular heat-stress has been shown to reduce adrenaline and cortisol, increase the cytotoxicity of NK cells, and enhance the proliferative response of B cells ( Tomiyama *et al.,* 2015). Heat-stress also stimulates the release of Heat Shock Proteins (HSPs), ( Iguchi *et al.,* 2012) which play an important role in antigen presentation and cross-presentation, activation of macrophages and lymphocytes, and activation and maturation of dendritic cells ( Tsan & Gao, 2009) as well as serving a chaperone function and protecting immune cells and proteins from heat-induced damage ( Singh & Hasday, 2013). The immunostimulatory effects of heat stress on the innate immune system may be further enhanced by alternating heat exposure with exposure to cold ( Heinonen & Laukkanen, 2018), which leads to leukocytosis, granulocytosis, an increase in NK cell count and activity, and an elevation in circulating levels of IL-6 ( Brenner *et al.,* 1999).

In addition to enhancing cellular responses, heat-stress induces a hormetic stress response that builds physiological resilience and confers tolerance to subsequent stress in a similar way to exercise ( Gálvez *et al.,* 2018). Heat stress improves respiratory function by reducing pulmonary congestion and increasing tidal volume, vital capacity, ventilation, and forced expiratory volume of the lungs ( Laitinen *et al.,* 1988), and improves cardiovascular function by modulating the autonomic nervous system, reducing inflammation, oxidative stress and blood pressure, increasing cardiac output, plasma volume and peripheral blood flow, and improving endothelial function, lipid profile and arterial compliance ( Heinonen & Laukkanen 2018; Kunutsor *et al.,* 2018; Laukkanen *et al.,* 2018; Laukkanen & Kunutsor, 2019).

Heat stress also aids in detoxification via sweating ( Crinnion, 2011) through which some toxic elements are preferentially excreted ( Genuis *et al.,* 2011). Furthermore, when heat stress is followed by cold exposure, blood is shunted to the internal organs, which induces a diuresis ( Epstein, 1978), and further aids in detoxification ( Cochrane, 2004). Heat-stress may offer a further advantage against respiratory viral infections by altering blood pH through hyperthermia-induced hyperventilation and subsequent respiratory alkalosis ( Tsuji *et al.,* 2016), which creates alkaline conditions that may be more favourable to host defenses. The ability of a transient alkaline environment to inhibit viral replication and reduce infectivity has been demonstrated with human coronavirus 229E, which has maximal infectivity in acid conditions ( Lamarre & Talbot, 1989), and with coronavirus MHV-A59, which undergoes conformational changes in the spike glycoprotein at a pH of 8 at 37°C degrees and leads to rapid and irreversible inactivation and loss of infectivity ( Sturman *et al.,* 1990).

In addition to offering physiological advantages in the battle against viral infection such as COVID-19, heat-based treatments can support mental wellness and confer many psychological benefits. Sauna bathing and other forms of heat therapy require time and effort devoted towards active relaxation that can help divert attention from anxiety-producing news and/or relieve boredom associated with social confinement. Sauna bathing also enhances sleep ( Hussain *et al.,* 2019), which further supports immune function ( Irwin & Opp, 2017). Engaging in an activity with an intended positive outcome can also impart feelings of control that may otherwise be lacking, and doing something that feels good and having positive expectations elicits the power of positive thought and the placebo effect or ‘remembered wellness’ ( Benson & Friedman, 1996). Furthermore, the exploration of heat-tolerance induces a 'forced-mindfulness' and a focus on the breath, which has additional physical and psychological benefits ( Black & Slavich, 2016). In a time of social distancing, saunas can also provide a way for close family members to come together in ways that have supported family cohesion for generations in Finland and other Nordic countries ( Mather & Kaups, 1963).

## Clinical applications and implications

There are a range of heat-based interventions that can be used alongside other personal hygiene measures to aid in overcoming COVID-19. For example, warming and humidifying indoor environments can prevent drying of the nasal mucosa, increase muco-ciliary clearance and nasal patency, and provide symptomatic relief ( Ophir & Elad, 1987). The direct application of heat to the upper airways, routinely or at the first signs of infection, may further serve to inhibit or deactivate virions in the place where they first lodge. This has been demonstrated in vitro with temperatures of 45°C for 20 minutes activating immune cells, releasing HSPs and suppressing rhinovirus multiplication by more than 90% ( Conti *et al.,* 1999). The inhalation of steam with added essential oils with anti-viral, decongestant, anxiolytic and other properties, may further assist in facilitating muco-ciliary clearance and reducing viral load as well as providing physical and psychological relief ( Ali *et al.,* 2015; Lee *et al.,* 2017).

Inducing mild heat-stress through the use of hot springs (balneotherapy), hot baths, saunas, steam-rooms and application of hot mud (pelotherapy), can be used to mimic fever and activate immune defenses. Enhanced immunity has been demonstrated with hyperthermia induced by traditional Finnish saunas ( Pilch *et al.,* 2013), far-infrared saunas ( Sobajima *et al.,* 2018), heated nano-mist ( Tomiyama *et al.,* 2015), hot baths ( Downing *et al.,* 1988; Kappel *et al.,* 1991; Tsuchiya *et al.,* 2003; Zellner *et al.,* 2002) and geothermal mineral water ( Uzunoglu *et al.,* 2017). The beneficial effects of heat-stress may be further potentiated by the traditional practice of alternating heat with exposure to cold, which may translate into greater resistance to viral infections as evidenced by a randomised controlled trial that found regular hot and cold showers reduced work absenteeism during an influenza outbreak ( Buijze *et al.,* 2016).

In recent years far-infrared (FIR) saunas have been used as an alternative to the traditional Finnish saunas. These saunas use infrared emitters without water or added humidity and generally run at lower temperatures than Finnish saunas. While the use of FIR saunas to treat viral infection has not been studied, FIR radiation is reported to deactivate single-strand RNA viruses ( Huang & Li, 2020) and FIR saunas have been shown to raise body temperature, induce hormetic stress responses and support host defenses ( Shemilt *et al.,* 2019). FIR saunas, along with other heat-based technologies may therefore also prove useful in treating patients with COVID-19.

There are currently no clinical protocols for using heat in the treatment of COVID-19, yet heat has a long history of traditional use, and traditional practices such as alternating hot and cold immersions, post-heat relaxation and use of essential oils can inform their development. While sauna bathing is generally well tolerated, heat-based clinical protocols are needed to design future studies and inform clinical practice in order to minimise the risk of cross-infection and reduce the incidence of adverse effects, which can range from burns, cramps, dizziness and fainting, to heat exhaustion, heat stroke and death. Such protocols must therefore consider contra-indications such as unstable angina, severe infection or high fever ( Hannuksela & Ellahham, 2001) along with factors such as age, weight, fitness, hydration status, co-morbidities and the use of alcohol or prescription drugs ( Leyk *et al.,* 2019). Hydro-therapy treatment plans also need to consider timing, temperature and humidity, as water is 25 times more conductive than air, making steam rooms tolerable at temperatures around 50°C while dry saunas are tolerated at temperatures above 100°C.

While the clinical application of heat has promise in the prevention and treatment of COVID-19, there are significant challenges in implementing heat-based therapies. The current pandemic has seen the fear of infection lead to the widespread closure of public facilities such as bathing facilities, commercial hot springs, spas, gymnasiums, hotels and fitness centers that offer saunas and heat treatments, and while some countries such as Finland have a large number of private saunas, in most other locations private sauna ownership is limited to people with high socio-economic status. Thus, if sauna bathing is to be widely implemented, public bathing and sauna facilities will need to re-open and adopt infection control measures similar to those for dealing with COVID-19 in other public facilities ( Liang, 2020).

Heat is one of oldest forms of microbial control and still remains one of the most common methods for controlling and eradicating pathogens. The temperatures achieved within a sauna are well within the range required for pathogen control and often exceed temperatures of 60°C for 30 min, 65°C for 15 min or 80°C for 1 min, which have been shown to reduce coronavirus infectivity by at least 4 log10 ( Kampf *et al.,* 2020). While the temperatures, humidity and times required to specifically deactivate SAR-CoV-2 *in vivo* are yet to be determined, the temperature within a sauna makes risk of cross infection in public sauna facilities highly unlikely.

Strategies for limiting cross infection have been recently developed for re-opening some of the 3000 hot springs that were recently closed in China ( Wang, 2020). There are also moves to reopen facilities as quarantine zones where people can undergo heat treatments during isolation or as places of respite for overworked medical staff. It may also be possible for saunas and steam rooms to be included within hospitals and rehabilitation facilities for use by both patients and staff. Furthermore, simple home-based protocols can provide guidance on using heat for people who are currently self-isolated in their homes or in quarantine. Such protocols could be evaluated using crowd-sourced, citizen-science platforms, which may help to develop, test and optimize treatment strategies for current and future pandemics.

## Conclusion

Heat is a cheap, convenient and widely accessible therapeutic modality with a long history of traditional use, yet it remains to be seen if heat can be effective in the treatment or prevention of COVID-19. The relatively low cost and wide availability of heat-based treatments, along with multiple mechanisms of action that include both physical and psychological dimensions, makes heat an attractive option for combating viral infections. The integration of these ancient forms of treatment with modern technology may lead to a greater integration of natural therapies in mainstream healthcare, with the potential to support the wellbeing of both patients and medical staff. This may also lead to a greater convergence between the healthcare and wellness industries, and the development of systems and activities that build the wellbeing and resilience of the wider community, thereby reducing the impact of future pandemics.

## Data availability

No data is associated with this work.

## References

[ref-1] Ali B Al-Wabel NA Shams S: Essential oils used in aromatherapy: A systemic review. *Asian Pac J Trop Biomed.* 2015;5(8):601–611. 10.1016/j.apjtb.2015.05.007

[ref-2] Benson H Friedman R: Harnessing the power of the placebo effect and renaming it "remembered wellness". *Annu Rev Med.* 1996;47:193–199. 10.1146/annurev.med.47.1.193 8712773

[ref-3] Black DS Slavich GM: Mindfulness meditation and the immune system: a systematic review of randomized controlled trials. *Ann N Y Acad Sci.* 2016;1373(1):13–24. 10.1111/nyas.12998 26799456 PMC4940234

[ref-4] Brenner IK Castellani JW Gabaree C: Immune changes in humans during cold exposure: effects of prior heating and exercise. *J Appl Physiol (1985).* 1999;87(2):699–710. 10.1152/jappl.1999.87.2.699 10444630

[ref-5] Buijze GA Sierevelt IN van der Heijden BC: The Effect of Cold Showering on Health and Work: A Randomized Controlled Trial. *PLoS One.* 2016;11(9):e0161749. 10.1371/journal.pone.0161749 27631616 PMC5025014

[ref-6] Chan KH Peiris JS Lam SY: The Effects of Temperature and Relative Humidity on the Viability of the SARS Coronavirus. *Adv Virol.* 2011;2011:734690. 10.1155/2011/734690 22312351 PMC3265313

[ref-60] Chicheł A Skowronek J Kubaszewska M: Hyperthermia – description of a method and a review of clinical applications. *Reports of Practical Oncology & Radiotherapy.* 2007;12(5):267–275. 10.1016/S1507-1367(10)60065-X

[ref-7] Clark-Kennedy J Cohen M: Indulgence or therapy? Exploring the characteristics, motivations and experiences of hot springs bathers in Victoria, Australia. *Asia Pacific Journal of Tourism Research.* 2017;22(5):501–511. 10.1080/10941665.2016.1276946

[ref-8] Cochrane DJ: Alternating hot and cold water immersion for athlete recovery: a review. *Physical Therapy in Sport.* 2004;5(1):26–32. 10.1016/j.ptsp.2003.10.002

[ref-9] Conti C De Marco A Mastromarino P: Antiviral effect of hyperthermic treatment in rhinovirus infection. *Antimicrob Agents Chemother.* 1999;43(4):822–829. 10.1128/aac.43.4.822 10103186 PMC89212

[ref-10] Crinnion WJ: Sauna as a valuable clinical tool for cardiovascular, autoimmune, toxicant- induced and other chronic health problems. *Altern Med Rev.* 2011;16(3):215–225. 21951023

[ref-11] Darnell ME Subbarao K Feinstone SM: Inactivation of the coronavirus that induces severe acute respiratory syndrome, SARS-CoV. *J Virol Methods.* 2004;121(1):85–91. 10.1016/j.jviromet.2004.06.006 15350737 PMC7112912

[ref-12] Downing JF Martinez-Valdez H Elizondo RS: Hyperthermia in humans enhances interferon-gamma synthesis and alters the peripheral lymphocyte population. *J Interferon Res.* 1988;8(2):143–150. 10.1089/jir.1988.8.143 3132509

[ref-13] Duan SM Zhao XS Wen RF: Stability of SARS coronavirus in human specimens and environment and its sensitivity to heating and UV irradiation. *Biomed Environ Sci.* 2003;16(3):246–255. 14631830

[ref-14] Epstein M: Renal effects of head-out water immersion in man: implications for an understanding of volume homeostasis. *Physiol Rev.* 1978;58(3):529–581. 10.1152/physrev.1978.58.3.529 356067

[ref-15] Ernst E Pecho E Wirz P: Regular sauna bathing and the incidence of common colds. *Ann Med.* 1990;22(4):225–227. 10.3109/07853899009148930 2248758

[ref-16] Evans SS Repasky EA Fisher DT: Fever and the thermal regulation of immunity: the immune system feels the heat. *Nat Rev Immunol.* 2015;15(6):335–349. 10.1038/nri3843 25976513 PMC4786079

[ref-17] Fahy JV Dickey BF: Airway mucus function and dysfunction. *N Engl J Med.* 2010;363(23):2233–2247. 10.1056/NEJMra0910061 21121836 PMC4048736

[ref-18] Gálvez I Torres-Piles S Ortega-Rincón E: Balneotherapy, Immune System, and Stress Response: A Hormetic Strategy? *Int J Mol Sci.* 2018;19(6): pii: E1687. 10.3390/ijms19061687 29882782 PMC6032246

[ref-19] Genuis SJ Birkholz D Rodushkin I: Blood, urine, and sweat (BUS) study: monitoring and elimination of bioaccumulated toxic elements. *Arch Environ Contam Toxicol.* 2011;61(2):344–357. 10.1007/s00244-010-9611-5 21057782

[ref-20] Gujrathi AB Ambulgekar V Handal A: Effect of Steam Inhalation on Nasal Mucociliary Clearance in Normal Individuals and Nasal Disease State. *Journal of Contemporary Medical Research.* 2016;3(5):1262–1264. Reference Source

[ref-21] Hannuksela ML Ellahham S: Benefits and risks of sauna bathing. *Am J Med.* 2001;110(2):118–126. 10.1016/S0002-9343(00)00671-9 11165553

[ref-61] Heinonen I Laukkanen JA: Effects of heat and cold on health, with special reference to Finnish sauna bathing. *Am J Physiol Regul Integr Comp Physiol.* 2018;314(5):R629–R638. 10.1152/ajpregu.00115.2017 29351426

[ref-22] Hu L Trefethen JM Zeng Y: Biophysical characterization and conformational stability of Ebola and Marburg virus-like particles. *J Pharm Sci.* 2011;100(12):5156–5173. 10.1002/jps.22724 21858822

[ref-23] Huang WH Li EJ: Instability of Nucleic Acids in Airborne Microorganisms under Far Infrared Radiation.Preprints.org.2020 10.20944/preprints202002.0332.v1

[ref-24] Hussain J Cohen M: Clinical Effects of Regular Dry Sauna Bathing: A Systematic Review. *Evid Based Complement Alternat Med.* 2018;2018:1857413. 10.1155/2018/1857413 29849692 PMC5941775

[ref-25] Hussain JN Greaves RF Cohen MM: A hot topic for health: Results of the Global Sauna Survey. *Complement Ther Med.* 2019;44:223–234. 10.1016/j.ctim.2019.03.012 31126560

[ref-56] Iguchi M Littmann AE Chang SH: Heat stress and cardiovascular, hormonal, and heat shock proteins in humans. *J Athl Train.* 2012;47(2):184–190. 10.4085/1062-6050-47.2.184 22488284 PMC3418130

[ref-26] Irwin MR Opp MR: Sleep Health: Reciprocal Regulation of Sleep and Innate Immunity. *Neuropsychopharmacology.* 2017;42(1):129–155. 10.1038/npp.2016.148 27510422 PMC5143488

[ref-27] Kampf G Voss A Scheithauer S: Inactivation of coronaviruses by heat. *J Hosp Infect.* 2020; pii: S0195-6701(20)30124-9. 10.1016/j.jhin.2020.03.025 32243951 PMC7271332

[ref-28] Kappel M Stadeager C Tvede N: Effects of *in vivo* hyperthermia on natural killer cell activity, *in vitro* proliferative responses and blood mononuclear cell subpopulations. *Clin Exp Immunol.* 1991;84(1):175–180. 10.1111/j.1365-2249.1991.tb08144.x 2015709 PMC1535372

[ref-29] Kudo E Song E Yockey LJ: Low ambient humidity impairs barrier function and innate resistance against influenza infection. *Proc Natl Acad Sci U S A.* 2019;116(22):10905–10910. 10.1073/pnas.1902840116 31085641 PMC6561219

[ref-30] Kunutsor SK Laukkanen T Laukkanen JA: Frequent sauna bathing may reduce the risk of pneumonia in middle-aged Caucasian men: The KIHD prospective cohort study. *Respir Med.* 2017a;132:161–163. 10.1016/j.rmed.2017.10.018 29229091

[ref-63] Kunutsor SK Laukkanen T Laukkanen JA: Sauna bathing reduces the risk of respiratory diseases: a long-term prospective cohort study. *Eur J Epidemiol.* 2017b;32(12):1107–1111. 10.1007/s10654-017-0311-6 28905164

[ref-64] Kunutsor SK Laukkanen T Laukkanen JA: Longitudinal associations of sauna bathing with inflammation and oxidative stress: the KIHD prospective cohort study. *Ann Med.* 2018;50(5):437–442. 10.1080/07853890.2018.1489143 29897261

[ref-65] Laitinen LA Lindqvist A Heino M: Lungs and ventilation in sauna. *Ann Clin Res.* 1988;20(4):244–248. 3218895

[ref-31] Lamarre A Talbot PJ: Effect of pH and temperature on the infectivity of human coronavirus 229E. *Can J Microbiol.* 1989;35(10):972–974. 10.1139/m89-160 2819602

[ref-66] Laukkanen JA Kunutsor SK: Is sauna bathing protective of sudden cardiac death? A review of the evidence. *Prog Cardiovasc Dis.* 2019;62(3):288–293. 10.1016/j.pcad.2019.05.001 31102597

[ref-67] Laukkanen JA Laukkanen T Kunutsor SK: Cardiovascular and Other Health Benefits of Sauna Bathing: A Review of the Evidence. *Mayo Clin Proc.* 2018;93(8):1111–1121. 10.1016/j.mayocp.2018.04.008 30077204

[ref-68] Laukkanen T Khan H Zaccardi F: Association Between Sauna Bathing and Fatal Cardiovascular and All-Cause Mortality Events. *JAMA Intern Med.* 2015;175(4):542–8. 10.1001/jamainternmed.2014.8187 25705824

[ref-32] Lee Mk Lim S Song JA: The effects of aromatherapy essential oil inhalation on stress, sleep quality and immunity in healthy adults: Randomized controlled trial. *Eur J Integr Med.* 2017;12:79–86. 10.1016/j.eujim.2017.04.009

[ref-33] Lelie PN Reesink HW Lucas CJ: Inactivation of 12 viruses by heating steps applied during manufacture of a hepatitis B vaccine. *J Med Virol.* 1987;23(3):297–301. 10.1002/jmv.1890230313 2828525 PMC7166500

[ref-69] Leyk D Hoitz J Becker C: Health Risks and Interventions in Exertional Heat Stress. *Deutsches Arzteblatt international.* 2019;116(31-32):537–544. 10.3238/arztebl.2019.0537 31554541 PMC6783627

[ref-34] Liang TE: Handbook of COVID-19 Preventionand Treatment China. The First Affiliated Hospital, Zhejiang University School of Medicine.2020 Reference Source

[ref-35] Mace TA Zhong L Kilpatrick C: Differentiation of CD8+ T cells into effector cells is enhanced by physiological range hyperthermia. *J Leukoc Biol.* 2011;90(5):951–962. 10.1189/jlb.0511229 21873456 PMC3206471

[ref-36] Mäkinen TM Juvonen R Jokelainen J: Cold temperature and low humidity are associated with increased occurrence of respiratory tract infections. *Respir Med.* 2009;103(3):456–462. 10.1016/j.rmed.2008.09.011 18977127

[ref-37] Mather C Kaups M: The Finnish Sauna: A cultural index to settlement. *A Assoc Am Geog.* 1963;53(4):494–504. Reference Source

[ref-38] Ophir D Elad Y: Effects of steam inhalation on nasal patency and nasal symptoms in patients with the common cold. *Am J Otolaryngol.* 1987;8(3):149–153. 10.1016/s0196-0709(87)80037-6 3303983

[ref-39] Pilch W Pokora I Szyguła Z: Effect of a single finnish sauna session on white blood cell profile and cortisol levels in athletes and non-athletes. *J Hum Kinet.* 2013;39:127–135. 10.2478/hukin-2013-0075 24511348 PMC3916915

[ref-40] Sajadi MM Habibzadeh P Vintzeleos A: Temperature and Latitude Analysis to Predict Potential Spread and Seasonality for COVID-19.2020 Reference Source 10.1001/jamanetworkopen.2020.11834 PMC7290414 32525550

[ref-41] Schieber AMP Ayres JS: Thermoregulation as a disease tolerance defense strategy. *Pathog Dis.* 2016;74(9); pii: ftw106. 10.1093/femspd/ftw106 27815313 PMC5975229

[ref-42] Shemilt R Bagabir H Lang C: Potential mechanisms for the effects of far-infrared on the cardiovascular system - a review. *Vasa.* 2019;48(4):303–312. 10.1024/0301-1526/a000752 30421656

[ref-43] Singh IS Hasday JD: Fever, hyperthermia and the heat shock response. *Int J Hyperthermia.* 2013;29(5):423–435. 10.3109/02656736.2013.808766 23863046

[ref-57] Sobajima M Nozawa T Ihori H: Repeated Low-Temperature Sauna Therapy Improves Cardiac and Exercise Capacity as well as Immune Competence in Patients with Heart Failure. *Circulation.* 2018;126(suppl_21):A10868 Reference Source

[ref-44] Soni B Nayak AK: Effect of inspiration cycle and ventilation rate on heat exchange in human respiratory airways. *J Therm Biol.* 2019;84:357–367. 10.1016/j.jtherbio.2019.07.026 31466774

[ref-45] Sturman LS Ricard CS Holmes KV: Conformational change of the coronavirus peplomer glycoprotein at pH 8.0 and 37 degrees C correlates with virus aggregation and virus-induced cell fusion. *J Virol.* 1990;64(6):3042–50. 10.1128/JVI.64.6.3042-3050.1990 2159562 PMC249489

[ref-46] Tomiyama C Watanabe M Honma T: The effect of repetitive mild hyperthermia on body temperature, the autonomic nervous system, and innate and adaptive immunity. *Biomed Res.* 2015;36(2):135–142. 10.2220/biomedres.36.135 25876664

[ref-58] Tsan MF Gao B: Heat shock proteins and immune system. *J Leukoc Biol.* 2009;85(6):905–910. 10.1189/jlb.0109005 19276179

[ref-47] Tsuchiya Y Shimizu T Tazawa T: Effects of hot deep seawater bathing on the immune cell distribution in peripheral blood from healthy young men. *Environ Health Prev Med.* 2003;8(5):161–165. 10.1007/BF02897909 21432093 PMC2723508

[ref-48] Tsuji B Hayashi K Kondo N: Characteristics of hyperthermia-induced hyperventilation in humans. *Temperature (Austin).* 2016;3(1):146–160. 10.1080/23328940.2016.1143760 27227102 PMC4879782

[ref-49] Tyrrell D Barrow I Arthur J: Local hyperthermia benefits natural and experimental common colds. *BMJ.* 1989;298(6683):1280–1283. 10.1136/bmj.298.6683.1280 2500196 PMC1836535

[ref-50] Tyrrell DA: Hot news on the common cold. *Annu Rev Microbiol.* 1988;42:35–47. 10.1146/annurev.mi.42.100188.000343 2849371

[ref-51] Uzunoglu E Yentur S Kayar AH: Effect of mild heat stress on heat shock protein 70 in a balneotherapy model. *Eur J Integr Med.* 2017;9(C):86–90. 10.1016/j.eujim.2016.11.014

[ref-52] Wang J: Crisis and Opportunities for the Chinese Hot Springs Industry in Year 2020.China, Asia-Pacific Institute for Hydrotherapy and Climatotherapy Tourism.2020 Reference Source

[ref-53] Wang J Tang K Feng K: High Temperature and High Humidity Reduce the Transmission of COVID-19.2020 Reference Source

[ref-54] WHO Report: First data on stability and resistance of SARS coronavirus compiled by members of WHO laboratory network, WHO Multi-center Collaborative Network on SARS Diagnosis.2003 Reference Source

[ref-55] Zellner M Hergovics N Roth E: Human monocyte stimulation by experimental whole body hyperthermia. *Wien Klin Wochenschr.* 2002;114(3):102–107. 12060966

