# Supplementary material for: Turning up the heat on COVID-19: heat as a therapeutic intervention
Source: F1000Res. 2020 Jul 20;9:292. Originally published 2020 Apr 24. [Version 2] doi: 10.12688/f1000research.23299.2 (PMC7372531; doi:10.12688/f1000research.23299.2)
Supplement: Peer review history

# Peer review: Reviewer response for version 2

**DOI:** 10.5256/f1000research.28030.r67436

**Published:** 2020-07-20

**Authors:** Jari Laukkanen

**Affiliations:** 1 Institute of Clinical Medicine, Department of Medicine, University of Eastern Finland, Kuopio, Finland

No other comments.

Is the topic of the opinion article discussed accurately in the context of the current literature?

Yes

Are arguments sufficiently supported by evidence from the published literature?

Yes

Are all factual statements correct and adequately supported by citations?

Yes

Are the conclusions drawn balanced and justified on the basis of the presented arguments?

Yes

Reviewer Expertise:

Cardiovascular diseases, sauna and exercise

I confirm that I have read this submission and believe that I have an appropriate level of expertise to confirm that it is of an acceptable scientific standard.

# Peer review: Reviewer response for version 1

**DOI:** 10.5256/f1000research.25720.r62786

**Published:** 2020-07-07

**Authors:** Jari Laukkanen

**Affiliations:** 1 Institute of Clinical Medicine, Department of Medicine, University of Eastern Finland, Kuopio, Finland

Heat is a cheap, convenient and widely accessible therapeutic modality. Sauna bathing is a form of heat therapy. However, there are no very accurate details of temperature, weekly frequency, and duration which would be needed to achieve those beneficial effects. An only traditional Finnish sauna is based on high temperatures.

 You should discuss more with the lights of many published original studies on sauna and its protective health effects. The literature review of this narrative has missed many recent papers on this topic. Please consider adding the recently published key references on sauna and health outcomes so that this article would be more up-to-date. ^1^^,^^2^^,^[ref-3 ^,^^4^^,^^5^^,^^6^^,^^7^

 What is the role of improved hygiene control due to regular sauna bathing?

 Warm and cold has any effect on the results?

 You could add studies that may indicate that sauna is related to inflammatory markers or cytokines. 

 It is written that emerging evidence suggests regular sauna bathing enhances cardiovascular function. No relevant references were available in the manuscript.

Is the topic of the opinion article discussed accurately in the context of the current literature?

Yes

Are arguments sufficiently supported by evidence from the published literature?

Yes

Are all factual statements correct and adequately supported by citations?

Yes

Are the conclusions drawn balanced and justified on the basis of the presented arguments?

Yes

Reviewer Expertise:

Cardiovascular diseases, sauna and exercise

I confirm that I have read this submission and believe that I have an appropriate level of expertise to confirm that it is of an acceptable scientific standard, however I have significant reservations, as outlined above.

## References

[1] : Cardiovascular and Other Health Benefits of Sauna Bathing: A Review of the Evidence. *Mayo Clin Proc*.93(8) : 10.1016/j.mayocp.2018.04.008 1111-1121 10.1016/j.mayocp.2018.04.008 30077204

[2] : Longitudinal associations of sauna bathing with inflammation and oxidative stress: the KIHD prospective cohort study. *Ann Med*.50(5) : 10.1080/07853890.2018.1489143 437-442 10.1080/07853890.2018.1489143 29897261

[3] : Effects of heat and cold on health, with special reference to Finnish sauna bathing. *American Journal of Physiology-Regulatory, Integrative and Comparative Physiology*.2018;314(5) : 10.1152/ajpregu.00115.2017 R629-R638 10.1152/ajpregu.00115.2017 29351426

[4] : Sauna bathing and systemic inflammation. *European Journal of Epidemiology*.2018;33(3) : 10.1007/s10654-017-0335-y 351-353 10.1007/s10654-017-0335-y 29209938

[5] : Sauna bathing reduces the risk of respiratory diseases: a long-term prospective cohort study. *European Journal of Epidemiology*.2017;32(12) : 10.1007/s10654-017-0311-6 1107-1111 10.1007/s10654-017-0311-6 28905164

[6] : Association between sauna bathing and fatal cardiovascular and all-cause mortality events. *JAMA Intern Med*.2015;175(4) : 10.1001/jamainternmed.2014.8187 542-8 10.1001/jamainternmed.2014.8187 25705824

[7] : Is sauna bathing protective of sudden cardiac death? A review of the evidence. *Prog Cardiovasc Dis*.62(3) : 10.1016/j.pcad.2019.05.001 288-293 10.1016/j.pcad.2019.05.001 31102597

# Peer review: Reviewer response for version 1

**DOI:** 10.5256/f1000research.25720.r62783

**Published:** 2020-05-13

**Authors:** Elizabeth A. Repasky

**Affiliations:** 1 Department of Immunology, Roswell Park Comprehensive Cancer Center, Buffalo, NY, USA

This is a timely, interesting, and generally well-written article summarizing many of the positive benefits of thermal therapies, provided in the context of potential therapies for patients with COVID-19. In general, the authors cover a wide range of literature related to applications (local/regional, whole body, topical) of elevated temperature. My comments related only to organization and some references and distinguishing better the difference between fever and hyperthermia. Heat treatments are likely to be hyperthermia treatments. While fever contains a thermal element, fever and hyperthermia represent very different physiological circumstances.   

 For example, statements such as that in Paragraph 2, right column, page3 "Fever has multiple actions with dealing with infections that include direct inhibition of pathogens, stimulation of both the innate and adaptive arms of the immune system and activation....". While the authors provide a review article as a reference here, most of this literature deals with hyperthermia and not actual infectious fever and this difference needs to be highlighted better. One would not be treated with fever. Moreover, there are other fundamental differences. Febrile individuals are usually quite cold and surface vessels constricted to conserve heat and help raise body temperature. Hyperthermia causes sweating and surface blood vessel dilation because the set point is not elevated.

 Moreover, there is a massive literature on heat stress/stroke (from hyperthermia, not fever) that can kill people because of excessive adhesion of platelets and white blood cells in capillaries. While the authors do rightly mention some safety concerns, it is perhaps a bit insufficient with regard to the impact of both hyperthermia (and fever). At the same time, the scope of the beneficial effects of mild elevation of body temperature and local temperature (in particular with respect to viral infections) is quite important to remember and hopefully expand upon in future clinical applications.

 Some topics should be highlighted more. For example, there is a substantial literature using electromagnetic heating and infrared heating for cancer therapy and so there is already safe equipment available for both local/regional as well as surface heating. Reviews on this topic have been available for some time. This technology could already be available for COVID-19 patients.  

 I found some of the referencing related to the direct impact of heat on viral replication (either in vivo (e.g., nasal cavities or in vitro) to be scattered a bit throughout the review. Some better reorganization of these various sections would help the reader to assess this very important point.

Is the topic of the opinion article discussed accurately in the context of the current literature?

Partly

Are arguments sufficiently supported by evidence from the published literature?

Yes

Are all factual statements correct and adequately supported by citations?

Yes

Are the conclusions drawn balanced and justified on the basis of the presented arguments?

Yes

Reviewer Expertise:

Immunology; Thermal stress; hyperthermia, anti-tumor immunity; Stress

I confirm that I have read this submission and believe that I have an appropriate level of expertise to confirm that it is of an acceptable scientific standard.
